# Terpene Synthase Gene Amplicons from Subseafloor Sediment

**DOI:** 10.1128/mra.01037-22

**Published:** 2023-01-18

**Authors:** Ruth L. Schmidt, Tatsuhiko Hoshino, Yuki Morono, Julien Tremblay, Dana Ulanova

**Affiliations:** a Centre Armand-Frappier Santé Biotechnologie, Institut national de la recherche scientifique, Laval, Québec, Canada; b Geomicrobiology Group, Kochi Institute for Core Sample Research, Institute for Extra-cutting-edge Science and Technology Avant-garde Research, Japan Agency for Marine-Earth Science and Technology (JAMSTEC), Nankoku, Kochi, Japan; c Energy, Mining and Environment Research Centre, National Research Council Canada, Montreal, Canada; d Department of Marine Resource Science, Faculty of Agriculture and Marine Science, Kochi University, Nankoku, Kochi, Japan; e Center for Advanced Marine Core Research, Kochi University, Nankoku, Kochi, Japan; University of Southern California

## Abstract

In this announcement, we present the set of putative terpene synthase (TS) gene fragments detected in a subseafloor sediment sample collected off Shimokita Peninsula, Japan. This data set contains sequences with 72 to 100% identity to TS from actinobacteria and cyanobacteria.

## ANNOUNCEMENT

Terpenoids (TRPs) are the largest class of specialized metabolites (1), and many of these compounds are known to act as signals in microbial interactions (2, 3).

Subseafloor sediments represent an environment with unique microbiological communities and metabolic activities (4). However, the knowledge of TRPs in the subseafloor environment remains limited. Here, we announce the detection of sequences with high similarity to terpene synthase (TS) genes of common bacterial TRPs, geosmin and 2-methylisoborneol (2-MIB) (3), in environmental DNA isolated from deep-sea subseafloor sediment.

The sediment sample was collected during the D/V *Chikyu* shakedown cruise of CK-06-06 (41.1771°N, 142.2016°E, 1,180 m, 5.2 m below the seafloor [mbsf]) and frozen at −80°C immediately after the sampling. DNA was extracted from 5 g of the frozen sediment as previously described (4, 5). In brief, DNA was extracted using DNeasy PowerMax soil kit (Qiagen) according to the manufacturer’s instruction with small modifications; concentrations were determined by PicoGreen (Thermo Fisher Scientific) after ethanol precipitation.

The geosmin TS fragment (432 bp) was amplified using primers geosmin-for (5′-*TCGTCGGCAGCGTCAGATGTGTATAAGAGACAG*CATCGAGATGCGSCGCAAGG-3′) and geosmin-rev (5′-*GTCTCGTGGGCTCGGAGATGTGTATAAGAGACAG*GASCGSAKGTGCCACTCGTG-3′) primers (adapter sequences are *in italics*). The 2-MIB TS primers were mib-for (5′-*TCGTCGGCAGCGTCAGATGTGTATAAGAGACAG*ACGACDNBTACTGCGAGGAC-3′) and mib-rev (5′-*GTCTCGTGGGCTCGGAGATGTGTATAAGAGACAG*GGVCGGAAGTTGTTGAACTG-3′) (331 bp). The PCR mix consisted of 1× EmeraldAmp Max PCR master mix (TaKaRa), 0.4 μM each primer, and 0.05 ng of sediment DNA. The two-phase touchdown PCR protocol for increased specificity and sensitivity was used (6). The cycling conditions, which were the same for both primer sets, were 95°C for 60 s, followed by 15 cycles of 98°C for 10 s, a touchdown gradient from 65°C to 50°C for 30 s, and 72°C for 30 s. The second phase was 20 cycles of 98°C for 10 s, 50°C for 30 s, and 72°C for 30 s. PCR products of expected sizes were excised from the agarose gel (NucleoSpin gel and PCR clean-up kit [Macherey-Nagel]) and purified using AMPure magnetic beads (Beckman Coulter). Twenty nanograms of each PCR product was used for index library preparation (Nextera XT index kit [Illumina] and Tks GFlex DNA polymerase [TaKaRa]). Libraries were purified as described above, quantified by QuantiFluor (Promega), and sequenced using 500-cycle MiSeq reagent nanokit v2 (MiSeq system, Illumina).

The obtained sequences (Table 1) were processed with the AmpliconTagger v1.3.0 pipeline (7). Default parameters were used for all software unless otherwise specified; details are available at https://zenodo.org/record/7455812. Raw reads were quality controlled and clustered at 97% identity to generate operational taxonomic units (OTUs). OTUs were filtered for chimeras using VSEARCH’s implementation of UCHIME *de novo* (8) and blasted against the NCBI nucleotide (nt) database (9). Hits with an E value of <1e-20, alignment length ≥100, and alignment percentage ≥60 were kept to build the RDP classifier (10) training set for an OTU taxonomic lineage assignment. Bacterial or archaeal lineages were combined with the OTU abundance matrix to generate a raw OTU table. The sequence-specific primer sequences were removed using MEGA v7.0.26 (11).

**TABLE 1:** Amplicon sequence counts and the blastx annotation results obtained in this study

Primer set	Total no. of reads	No. of reads clustered in OTUs (clusters ≥10 reads)	Total no. of OTUs	No. of TS-annotated OTUs (no. of reads)^*a*^	Highest blastx hit^*b*^	% identity
Geosmin TS	122,670	35,055	39	23 (32,826)	Actinobacterial/cyanobacterial geosmin and terpene synthases	72–100
2-MIB TS	42,472	10,363	17	10 (10,269)	Streptomycete cyclases/2-MIB synthases	86–98

a OTUs with the highest hits to TS sequences as annotated by blastx algorithm (12) and NCBI GenBank nonredundant (nr) protein sequence database (accessed October to November 2020). Read numbers of TS-annotated OTUs are given in brackets.

b The highest blastx hits of TS-annotated OTUs from the “no. of TS-annotated OTUs (no. of reads)” column.

This data set can be used in studies on TS gene diversity and distribution in subseafloor environments.

### Data availability.

Raw reads were deposited in a BioProject at DDBJ/ENA/GenBank under the accession number PRJNA846928. The GenBank accession numbers for OTUs are ON723903 to ON723912 (2-MIB) and ON723913 to ON723935 (geosmin).
